# Crystal structure of silver [(propane-1,3-diyl­dinitrilo-κ^2^ *N*,*N*′)­tetra­acetato-κ^4^ *O*,*O*′,*O*′′,*O*′′′]chromate(III) from synchrotron X-ray data

**DOI:** 10.1107/S2056989018001743

**Published:** 2018-02-02

**Authors:** Dohyun Moon, Keon Sang Ryoo, Jong-Ha Choi

**Affiliations:** a Pohang Accelerator Laboratory, POSTECH, Pohang 37673, Republic of Korea; b Department of Chemistry, Andong National University, Andong 36729, Republic of Korea

**Keywords:** crystal structure, propane-1,3-diyldi­nitrilo­tetra­acetate, silver cation, chro­mate(III) complex, twist-boat conformer

## Abstract

The asymmetric unit of the title compound, Ag[Cr(C_11_H_14_N_2_O_8_)]·3H_2_O, contains one [Cr(1,3-pdta)]^−^ anion [1,3-pdta is (propane-1,3-diyldi­nitrilo)­tetra­acetate], one Ag^+^ cation and three water mol­ecules. The Cr^3+^ ion is coordinated to the four O and two N atoms of the 1,3-pdta ligand, displaying a distorted octa­hedral geometry. The mean Cr—N and Cr—O bond lengths are 2.0727 (17) and 1.9608 (15) Å, respectively. The conformations of the chelate rings were found to be envelope for the glycinates and twist-boat for the six-membered di­amine (*T*) ring. The Ag^+^ cation is surrounded by six O atoms from three non-coordinated carbonyl O atoms of neighbouring 1,3-pdta groups and three water mol­ecules. The crystal structure is stabilized by inter­molecular hydrogen bonding involving the water O—H group as donor and the carboxyl O atom as acceptor.

## Abstract 2

In the title complex, the Cr^3+^ ion is coordinated by the four O and two N atoms of the 1,3-pdta ligand, displaying a distorted octa­hedral geometry. The Ag^+^ cation is surrounded by six O atoms from neighboring 1,3-pdta groups and water mol­ecules.

## Chemical context   

The hexa­dentate ligand, propane-1,3-diyldi­nitrilo­tetra­acetate (abbreviated here as 1,3-pdta, C_11_H_14_N_2_O_8_) has been used for the preparation of complexes with many transition metal ions (Herak *et al.*, 1984 ▸; Yamamoto *et al.*, 1988 ▸; Douglas & Radanović, 1993 ▸). In the complex anion, [*M(*1,3-pdta)]^n-^, the six-membered propane-1,3-di­amine ring is referred to as the *T* ring, the equatorially coordinated glycinate ring as the *G* ring, and the axially coordinated glycinate ring as the *R* ring (see Scheme). The counter-ion and metal-center oxidation state play a very important role in conformational isomerism. Upon coordination of 1,3-pdta by a metal center, the six-membered *T* ring can take twist-boat or half-chair conformers (Meier *et al.*, 2007 ▸). The twist-boat conformer was found in the crystal structures of K[Co(1,3-pdta)]·2H_2_O (Nagao *et al.*, 1972 ▸), Li[Fe(1,3-pdta)]·3H_2_O (Yamamoto *et al.*, 1988 ▸) and Na[Cr(1,3-pdta)]·3H_2_O (Herak *et al.*, 1984 ▸), whereas the half-chair form was observed in structural studies of [C(NH_2_)_3_][Fe(1,3-pdta)]·2H_2_O (Meier *et al.*, 2007 ▸) and Li_2_[Co(1,3-pdta)]·3H_2_O (Rychlewska *et al.*, 2008 ▸). The crystal structure of Na[Cr(1,3-pdta)]·3H_2_O (Herak *et al.*, 1984 ▸) has also been reported previously. In this communication, we report the crystal structure of Ag[Cr(1,3-pdta)]·3H_2_O in order to clarify unambiguously the bonding mode and the conformational geometry adopted by the Ag^+^ salt.
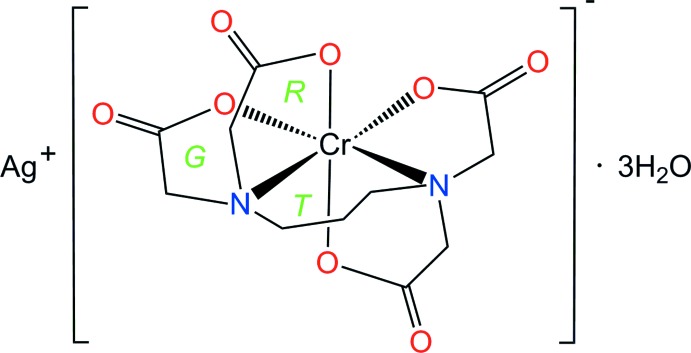


## Structural commentary   

**Figure 1:**
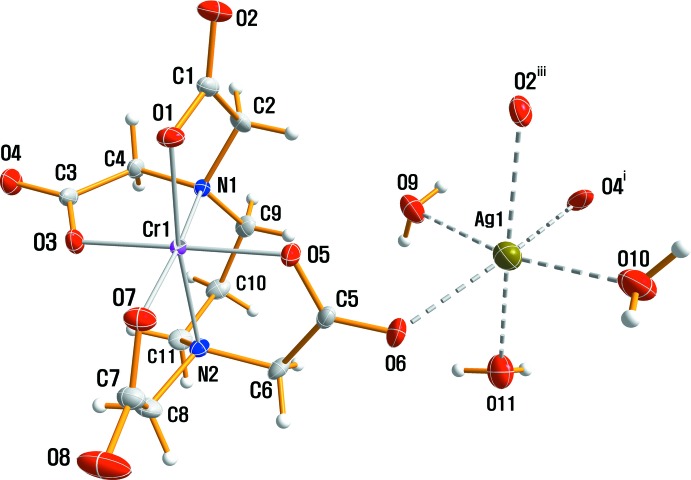
The structures of the mol­ecular entities in compound (I), showing the atom-numbering scheme. Non-H atoms are shown with displacement ellipsoids at the 50% probability level. [Symmetry codes: (i) −*x* + 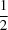, −*y* + 1, *z* + 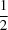; (iii) *x* + 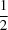, −*y* + 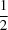, 1 − *z*.]

This is another example of a [Cr(1,3-pdta)]^−^ anion but with a different cation. The crystal structure of the title compound is isotypic with Na[*M*(1,3-pdta)]·3H_2_O (*M* = Fe, Cr or Rh; Okamoto *et al.*, 1990 ▸; Herak *et al.*, 1984 ▸) but it belongs to the ortho­rhom­bic space group *P*2_1_2_1_2_1_ compared with the monoclinic space group *P*2_1_/*n* of Li[Fe(1,3-pdta)]·3H_2_O (Yamamoto *et al.*, 1988 ▸) and ortho­rhom­bic space group *B*22_1_2 of K[Co(1,3-pdta)]·2H_2_O (Nagao *et al.*, 1972 ▸). The structural analysis shows that the propane-1,3-diyldi­nitrilo­tetra­acetate anion is coord­in­ated octa­hedrally by the Cr metal center through four O and two N atoms. An ellipsoid plot of title complex showing the atomic numbering is given in Fig. 1 ▸. The Cr—O bond distances differ slightly, the mean equatorial and axial distances being 1.9672 (15) and 1.9544 (15) Å, respectively. The *cis* angles at the Cr^III^ ion range from 81.66 (6) to 99.41 (6)° and the *trans* angles are 173.07 (7), 175.01 (6) and 176.04 (7)°. The six-membered propane-1,3-di­amine *T* ring (Fig. 1 ▸) adopts a flexible twist-boat conformation. The *R* rings are nearly planar and are in an envelope conformation. The *G* rings are much more puckered and are halfway between an envelope and a twist conformation. The Cr—O bond distances are greater in the *G* rings than in the *R* rings, and the average Cr—N bond length of 2.0727 (17) Å is 0.1119 Å longer than the average Cr—O bond distance. The Cr—N and Cr—O bond distances are in accordance with the values observed in Na[Cr(1,3-pdta)]·3H_2_O. However, the average Ag—O distance of 2.525 (2) Å is slightly longer than the Na—O distance of 2.437 Å in Na[Cr(1,3-pdta)]·3H_2_O (Herak *et al.*, 1984 ▸).

## Supra­molecular features   

**Figure 2:**
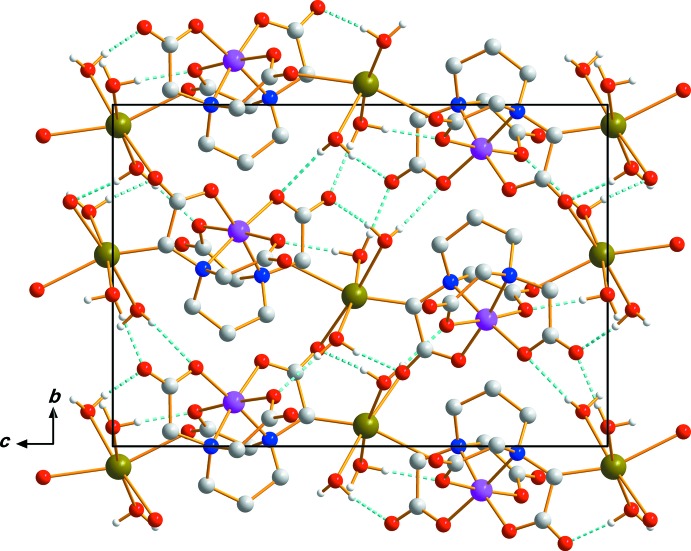
Crystal packing of Ag[Cr(1,3-pdta)]·3H_2_O, viewed perpendicular to the *bc* plane. Dashed lines represent O—H⋯O hydrogen-bonding inter­actions.

The Ag^+^ cation is surrounded octa­hedrally by three water mol­ecules (O9, O10 and O11) and three carboxyl­ate O atoms [O6, O2^iii^(*x* + 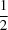, −*y* + 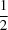, 1 − *z*) and O4^i^(−*x* + 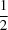, −*y* + 1, *z* + 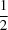)] that are not directly coordinated to the Cr atom (Fig. 1 ▸). Hydrogen bonds exist between the water mol­ecules and the O atoms in the 1,3-pdta moiety (Table 1 ▸). An extensive array of these contacts generate a three-dimensional network of mol­ecules stacked along the *a*-axis direction (Fig. 2 ▸). Non-coord­inating and coordinating carboxyl­ate O atoms take part in the formation of O—H⋯O hydrogen bonds, which contribute to the crystal packing stabilization and give rise to an infinite three-dimensional framework.

## Database survey   

A search of the Cambridge Structural Database (Version 5.38, May 2017 with three updates; Groom *et al.*, 2016 ▸) gave just three hits for a related complex anion, the [Cr(C_11_H_14_N_2_O_8_)_2_]^−^ unit. The crystal structure with an Na^+^ counter-cation (Herak *et al.*, 1981 ▸, 1984 ▸) has been determined. The crystal structures of Na[Cr(1,3-pndta)]·H_2_O, K[Cr(1,3-pndta)]·H_2_O and Ca[Cr(1,3-pndta)]_2_·4H_2_O (1,3-pndta = pentane-1,3-diyldi­nitrilo­tetra­acetate; Warżajtis *et al.*, 2014 ▸) have been reported previously. However, no structure of a [Cr(1,3-pdta)]^−^ or [Cr(1,3-pndta)]^−^ complex with Ag^+^ cation was found.

## Synthesis and physical measurements   

All chemicals were reagent-grade materials and were used without further purification. The UV–Vis absorption spectrum was recorded with a Cary 5000 UV–Vis–NIR Spectrophotometer. The FT–IR spectrum was obtained from a KBr pellet with a JASCO 460 plus series FT–IR spectrometer. Analyses for C, H, N were performed on a Carlo Erba 1108 Elemental Vario EL analyser. The precursor salt, Na[Cr(1,3-pdta)]·3H_2_O was prepared as described previously (Weyh & Hamm, 1968 ▸; Herak *et al.*, 1984 ▸). The sodium salt (0.20 g) was dissolved in 15 mL of water at 323 K and added to 3 mL of water containing 0.5 g of AgNO_3_. The resulting solution was filtered and left to stand at room temperature for several days to give purple block-shaped crystals of the silver salt, Ag[Cr(1,3-pdta)]·3H_2_O suitable for X-ray structural analysis. Elemental analysis calculated for Ag[Cr(C_11_H_14_N_2_O_8_)]·3H_2_O: C, 25.60; H, 3.91; N, 5.43%; found: C, 25.71; H, 3.23; N, 5.36%. UV–vis data (H_2_O solution, nm): 201 (*vs*), 223 (*vs*), 245 (*sh*), 385 (*s*), 506 (*s*), 700 (*w*). IR spectrum (KBr, cm^−1^) : 3447 (*vs, br*) (*ν* OH), 3232 (*sh*), 2977 (*vs*) and 2941 (*s*) (*ν* CH), 1643 (*s, br*) (*ν_as_* COO), 1473 (*s*), 1428 (*m*), 1363 (*vs*) and 1327 (*vs*) (*ν_s_* COO), 1271 (*sh*), 1222 (*s*), 1144 (*s*), 1099 (*vs*), 1061 (*m*), 1029 (*s*), 988 (*s*), 941 (*vs*), 916 (*vs*), 897 (*m*), 853 (*vs*), 746 (*vs*), 690 (*m*), 632 (*w*), 579 (*m*), 529 (*s*), 486 (*s*), 433 (*s*).

## Refinement   

Crystal data, data collection and structure refinement details are summarized in Table 2 ▸. C-bound H atoms were placed in geometrically idealized positions and constrained to ride on their parent atoms, with C—H = 0.97 Å with *U*_iso_(H) = 1.2*U*_eq_(C). O-bound H atoms were assigned based on a difference-Fourier map, and were refined with distance restraints of 0.88 (2) Å (using DFIX and DANG commands), and *U*_iso_(H) = 1.2*U*_eq_(O).

## Supplementary Material

Crystal structure: contains datablock(s) I. DOI: 10.1107/S2056989018001743/nk2244sup1.cif

Structure factors: contains datablock(s) I. DOI: 10.1107/S2056989018001743/nk2244Isup2.hkl

CCDC reference: 1424813

Additional supporting information:  crystallographic information; 3D view; checkCIF report

## Figures and Tables

**Table 1:** Hydrogen-bond geometry (Å, °)

*D*—H⋯*A*	*D*—H	H⋯*A*	*D*⋯*A*	*D*—H⋯*A*
O9—H1*O*1⋯O3^i^	0.84 (1)	1.95 (1)	2.797 (2)	178 (3)
O9—H2*O*1⋯O8^ii^	0.85 (1)	1.93 (1)	2.767 (3)	172 (4)
O10—H1*O*2⋯O5^iii^	0.85 (1)	2.02 (1)	2.870 (2)	173 (4)
O10—H2*O*2⋯O2^iv^	0.85 (1)	1.89 (1)	2.729 (3)	170 (4)
O11—H1*O*3⋯O7^ii^	0.84 (1)	2.33 (2)	3.142 (3)	163 (4)
O11—H2*O*3⋯O8^v^	0.83 (1)	1.99 (2)	2.791 (3)	161 (3)

Symmetry codes: (i) 
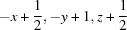
; (ii) 
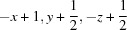
; (iii) 
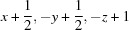
; (iv) 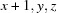; (v) 
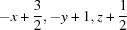
.

**Table 2:** Experimental details

Crystal data	
Chemical formula	Ag[Cr(C_11_H_14_N_2_O_8_)]·3H_2_O
*M*_r_	516.16
Crystal system, space group	Orthorhombic, *P*2_1_2_1_2_1_
Temperature (K)	260
*a*, *b*, *c* (Å)	8.7800 (18), 11.443 (2), 16.573 (3)
*V* (Å^3^)	1665.1 (6)
*Z*	4
Radiation type	Synchrotron, λ = 0.610 Å
μ (mm^−1^)	1.25
Crystal size (mm)	0.17 × 0.13 × 0.07

Data collection	
Diffractometer	ADSC Q210 CCD area detector
Absorption correction	Empirical (using intensity measurements) (*HKL3000sm *SCALEPACK**; Otwinowski & Minor, 1997 ▸)
*T*_min_, *T*_max_	0.843, 1.000
No. of measured, independent and observed [*I* > 2σ(*I*)] reflections	14937, 4807, 4738
*R*_int_	0.041
(sin θ/λ)_max_ (Å^−1^)	0.706

Refinement	
*R*[*F* ^2^ > 2σ(*F* ^2^)], *wR*(*F* ^2^), *S*	0.020, 0.051, 1.07
No. of reflections	4807
No. of parameters	253
No. of restraints	9
H-atom treatment	H atoms treated by a mixture of independent and constrained refinement
Δρ_max_, Δρ_min_ (e Å^−3^)	0.41, −0.65
Absolute structure	Flack *x* determined using 2027 quotients [(*I* ^+^)−(*I* ^−^)]/[(*I* ^+^)+(*I* ^−^)] (Parsons et al., 2013 ▸)
Absolute structure parameter	−0.008 (6)

Computer programs: *PAL BL2D-SMDC* (Shin *et al.*, 2016 ▸), *HKL3000sm* (Otwinowski & Minor, 1997 ▸), *SHELXT2014* (Sheldrick, 2015*a* ▸), *SHELXL2016* (Sheldrick, 2015*b* ▸), *DIAMOND* (Putz & Brandenburg, 2014 ▸) and *publCIF* (Westrip, 2010 ▸).

## supplementary crystallographic information

### Crystal data

**Table:**

Ag[Cr(C_11_H_14_N_2_O_8_)]·3H_2_O	*D*_x_ = 2.059 Mg m^−^^3^
*M**_r_* = 516.16	Synchrotron radiation, λ = 0.610 Å
Orthorhombic, *P*2_1_2_1_2_1_	Cell parameters from 33074 reflections
*a* = 8.7800 (18) Å	θ = 0.4–33.7°
*b* = 11.443 (2) Å	µ = 1.25 mm^−^^1^
*c* = 16.573 (3) Å	*T* = 260 K
*V* = 1665.1 (6) Å^3^	Block, purple
*Z* = 4	0.17 × 0.13 × 0.07 mm
*F*(000) = 1036	

### Data collection

**Table:**

ADSC Q210 CCD area detector diffractometer	4738 reflections with *I* > 2σ(*I*)
Radiation source: PLSII 2D bending magnet	*R*_int_ = 0.041
ω scan	θ_max_ = 25.5°, θ_min_ = 2.1°
Absorption correction: empirical (using intensity measurements) (*HKL3000sm Scalepack*; Otwinowski & Minor, 1997)	*h* = −12→12
*T*_min_ = 0.843, *T*_max_ = 1.000	*k* = −16→16
14937 measured reflections	*l* = −23→23
4807 independent reflections	

### Refinement

**Table:**

Refinement on *F*^2^	Hydrogen site location: mixed
Least-squares matrix: full	H atoms treated by a mixture of independent and constrained refinement
*R*[*F*^2^ > 2σ(*F*^2^)] = 0.020	*w* = 1/[σ^2^(*F*_o_^2^) + (0.0272*P*)^2^ + 0.5713*P*] where *P* = (*F*_o_^2^ + 2*F*_c_^2^)/3
*wR*(*F*^2^) = 0.051	(Δ/σ)_max_ = 0.002
*S* = 1.07	Δρ_max_ = 0.41 e Å^−^^3^
4807 reflections	Δρ_min_ = −0.65 e Å^−^^3^
253 parameters	Absolute structure: Flack *x* determined using 2027 quotients [(*I*^+^)-(*I*^-^)]/[(*I*^+^)+(*I*^-^)] (Parsons et al., 2013)
9 restraints	Absolute structure parameter: −0.008 (6)

### Special details

**Table:**

Geometry. All esds (except the esd in the dihedral angle between two l.s. planes) are estimated using the full covariance matrix. The cell esds are taken into account individually in the estimation of esds in distances, angles and torsion angles; correlations between esds in cell parameters are only used when they are defined by crystal symmetry. An approximate (isotropic) treatment of cell esds is used for estimating esds involving l.s. planes.

### Fractional atomic coordinates and isotropic or equivalent isotropic displacement parameters (Å^2^)

**Table:**

	*x*	*y*	*z*	*U*_iso_*/*U*_eq_	
Cr1	0.24189 (3)	0.37102 (2)	0.24502 (2)	0.00967 (6)	
O1	0.12226 (18)	0.25175 (12)	0.30121 (9)	0.0175 (3)	
O2	−0.0055 (2)	0.21547 (15)	0.41423 (11)	0.0251 (3)	
O3	0.08051 (18)	0.39909 (14)	0.16628 (9)	0.0186 (3)	
O4	−0.16003 (19)	0.45764 (18)	0.15293 (11)	0.0282 (4)	
O5	0.40372 (17)	0.35453 (13)	0.32531 (9)	0.0177 (3)	
O6	0.63816 (19)	0.40881 (18)	0.35973 (11)	0.0286 (4)	
O7	0.35275 (18)	0.27005 (12)	0.16811 (9)	0.0184 (3)	
O8	0.5114 (3)	0.27410 (18)	0.06396 (14)	0.0398 (5)	
N1	0.11336 (19)	0.48076 (14)	0.31737 (10)	0.0116 (3)	
N2	0.38257 (19)	0.50019 (14)	0.19855 (11)	0.0132 (3)	
C1	0.0637 (2)	0.28284 (17)	0.36905 (12)	0.0150 (3)	
C2	0.0876 (2)	0.41046 (17)	0.39202 (12)	0.0165 (3)	
H21	−0.001299	0.439533	0.420408	0.020*	
H22	0.174899	0.417403	0.427556	0.020*	
C3	−0.0428 (2)	0.45009 (19)	0.19226 (12)	0.0163 (3)	
C4	−0.0348 (2)	0.50289 (17)	0.27589 (13)	0.0155 (3)	
H41	−0.050662	0.586572	0.271860	0.019*	
H42	−0.116537	0.470866	0.308456	0.019*	
C5	0.5249 (2)	0.41833 (17)	0.31713 (12)	0.0159 (3)	
C6	0.5206 (2)	0.50999 (17)	0.25100 (15)	0.0177 (3)	
H61	0.522708	0.587001	0.275442	0.021*	
H62	0.611006	0.502164	0.217770	0.021*	
C7	0.4341 (3)	0.32390 (19)	0.11558 (13)	0.0192 (4)	
C8	0.4251 (3)	0.45637 (18)	0.11673 (13)	0.0204 (4)	
H81	0.522910	0.488802	0.101292	0.024*	
H82	0.349954	0.482279	0.077731	0.024*	
C9	0.1955 (2)	0.59037 (17)	0.33877 (13)	0.0183 (4)	
H91	0.286801	0.569504	0.368393	0.022*	
H92	0.131325	0.635424	0.374865	0.022*	
C10	0.2416 (3)	0.66904 (17)	0.26797 (15)	0.0214 (4)	
H10	0.153858	0.716501	0.254216	0.026*	
H10B	0.319794	0.721905	0.287443	0.026*	
C11	0.3006 (2)	0.61414 (17)	0.18957 (14)	0.0189 (4)	
H11	0.214770	0.602452	0.153619	0.023*	
H11B	0.368853	0.669379	0.163804	0.023*	
Ag1	0.66176 (2)	0.43997 (2)	0.51158 (2)	0.02983 (6)	
O9	0.4276 (2)	0.55599 (18)	0.50044 (10)	0.0323 (4)	
H1O1	0.426 (5)	0.571 (3)	0.5503 (8)	0.039*	
H2O1	0.455 (4)	0.6206 (19)	0.4800 (19)	0.039*	
O10	0.8668 (3)	0.31407 (19)	0.54800 (12)	0.0345 (4)	
H1O2	0.869 (5)	0.264 (3)	0.5862 (15)	0.041*	
H2O2	0.899 (4)	0.276 (3)	0.5075 (14)	0.041*	
O11	0.8203 (3)	0.60117 (17)	0.44997 (13)	0.0333 (4)	
H1O3	0.758 (4)	0.637 (3)	0.421 (2)	0.040*	
H2O3	0.853 (4)	0.649 (3)	0.4838 (18)	0.040*	

### Atomic displacement parameters (Å^2^)

**Table:**

	*U*^11^	*U*^22^	*U*^33^	*U*^12^	*U*^13^	*U*^23^
Cr1	0.00884 (12)	0.01014 (11)	0.01002 (11)	−0.00089 (9)	0.00008 (9)	0.00054 (9)
O1	0.0211 (7)	0.0136 (6)	0.0177 (6)	−0.0061 (5)	0.0050 (5)	−0.0013 (5)
O2	0.0265 (8)	0.0234 (7)	0.0253 (8)	−0.0017 (6)	0.0088 (7)	0.0093 (6)
O3	0.0137 (6)	0.0295 (7)	0.0127 (6)	0.0031 (5)	−0.0030 (5)	−0.0026 (5)
O4	0.0137 (6)	0.0496 (10)	0.0212 (7)	0.0015 (7)	−0.0065 (6)	0.0017 (7)
O5	0.0133 (6)	0.0219 (7)	0.0180 (6)	−0.0021 (5)	−0.0042 (5)	0.0052 (5)
O6	0.0154 (7)	0.0474 (10)	0.0230 (8)	−0.0030 (6)	−0.0080 (6)	0.0007 (7)
O7	0.0211 (7)	0.0143 (6)	0.0199 (7)	0.0007 (5)	0.0072 (6)	−0.0012 (5)
O8	0.0520 (13)	0.0286 (9)	0.0388 (11)	−0.0050 (9)	0.0299 (10)	−0.0106 (8)
N1	0.0116 (6)	0.0113 (6)	0.0119 (6)	−0.0005 (5)	−0.0009 (5)	−0.0003 (5)
N2	0.0117 (7)	0.0118 (7)	0.0160 (7)	−0.0009 (5)	0.0001 (5)	0.0022 (5)
C1	0.0140 (8)	0.0163 (8)	0.0147 (8)	−0.0005 (6)	0.0008 (6)	0.0039 (6)
C2	0.0206 (9)	0.0182 (8)	0.0108 (7)	0.0008 (7)	0.0019 (7)	0.0011 (6)
C3	0.0114 (8)	0.0233 (8)	0.0144 (8)	−0.0021 (7)	−0.0017 (6)	0.0044 (7)
C4	0.0110 (7)	0.0192 (9)	0.0163 (8)	0.0017 (6)	−0.0018 (6)	0.0003 (6)
C5	0.0110 (7)	0.0210 (8)	0.0157 (8)	−0.0002 (6)	−0.0018 (6)	−0.0034 (7)
C6	0.0108 (7)	0.0167 (7)	0.0258 (9)	−0.0031 (6)	−0.0016 (7)	0.0007 (7)
C7	0.0201 (9)	0.0205 (8)	0.0169 (9)	−0.0021 (7)	0.0049 (7)	−0.0025 (7)
C8	0.0253 (10)	0.0191 (9)	0.0167 (8)	−0.0036 (7)	0.0074 (8)	0.0019 (7)
C9	0.0203 (9)	0.0147 (7)	0.0200 (9)	−0.0028 (6)	−0.0008 (7)	−0.0056 (6)
C10	0.0201 (9)	0.0121 (7)	0.0319 (10)	−0.0014 (7)	0.0024 (8)	0.0002 (7)
C11	0.0215 (9)	0.0121 (8)	0.0230 (9)	0.0017 (6)	0.0009 (7)	0.0052 (7)
Ag1	0.03046 (10)	0.03290 (9)	0.02614 (9)	0.00309 (7)	0.00069 (7)	0.00446 (7)
O9	0.0440 (10)	0.0339 (8)	0.0191 (8)	−0.0016 (8)	−0.0045 (7)	0.0039 (7)
O10	0.0401 (11)	0.0378 (10)	0.0257 (9)	0.0108 (8)	0.0103 (8)	0.0094 (7)
O11	0.0376 (10)	0.0302 (8)	0.0320 (9)	0.0014 (8)	−0.0099 (8)	−0.0045 (7)

### Geometric parameters (Å, º)

**Table:**

Cr1—O3	1.9530 (15)	C4—H41	0.9700
Cr1—O5	1.9558 (15)	C4—H42	0.9700
Cr1—O1	1.9578 (14)	C5—C6	1.517 (3)
Cr1—O7	1.9766 (15)	C6—H61	0.9700
Cr1—N1	2.0708 (17)	C6—H62	0.9700
Cr1—N2	2.0745 (16)	C7—C8	1.518 (3)
O1—C1	1.287 (2)	C8—H81	0.9700
O2—C1	1.234 (2)	C8—H82	0.9700
O3—C3	1.303 (2)	C9—C10	1.533 (3)
O4—C3	1.222 (2)	C9—H91	0.9700
O5—C5	1.298 (2)	C9—H92	0.9700
O6—C5	1.224 (3)	C10—C11	1.533 (3)
O6—Ag1	2.5501 (19)	C10—H10	0.9700
O7—C7	1.284 (3)	C10—H10B	0.9700
O8—C7	1.232 (3)	C11—H11	0.9700
N1—C9	1.490 (2)	C11—H11B	0.9700
N1—C4	1.493 (2)	Ag1—O10	2.383 (2)
N1—C2	1.493 (2)	Ag1—O9	2.455 (2)
N2—C8	1.493 (3)	Ag1—O11	2.526 (2)
N2—C6	1.496 (3)	O9—H1O1	0.844 (13)
N2—C11	1.497 (3)	O9—H2O1	0.848 (13)
C1—C2	1.524 (3)	O10—H1O2	0.854 (13)
C2—H21	0.9700	O10—H2O2	0.847 (13)
C2—H22	0.9700	O11—H1O3	0.839 (13)
C3—C4	1.514 (3)	O11—H2O3	0.834 (13)
			
O3—Cr1—O5	176.04 (7)	O6—C5—C6	119.87 (19)
O3—Cr1—O1	92.48 (7)	O5—C5—C6	116.36 (17)
O5—Cr1—O1	89.94 (7)	N2—C6—C5	112.83 (16)
O3—Cr1—O7	91.29 (7)	N2—C6—H61	109.0
O5—Cr1—O7	91.41 (7)	C5—C6—H61	109.0
O1—Cr1—O7	99.41 (6)	N2—C6—H62	109.0
O3—Cr1—N1	83.79 (7)	C5—C6—H62	109.0
O5—Cr1—N1	93.47 (7)	H61—C6—H62	107.8
O1—Cr1—N1	81.66 (6)	O8—C7—O7	123.7 (2)
O7—Cr1—N1	175.01 (7)	O8—C7—C8	120.0 (2)
O3—Cr1—N2	93.82 (7)	O7—C7—C8	116.21 (18)
O5—Cr1—N2	83.61 (7)	N2—C8—C7	111.09 (16)
O1—Cr1—N2	173.07 (7)	N2—C8—H81	109.4
O7—Cr1—N2	83.33 (7)	C7—C8—H81	109.4
N1—Cr1—N2	96.16 (7)	N2—C8—H82	109.4
C1—O1—Cr1	115.94 (12)	C7—C8—H82	109.4
C3—O3—Cr1	117.10 (13)	H81—C8—H82	108.0
C5—O5—Cr1	118.06 (13)	N1—C9—C10	116.11 (17)
C5—O6—Ag1	128.51 (15)	N1—C9—H91	108.3
C7—O7—Cr1	115.52 (13)	C10—C9—H91	108.3
C9—N1—C4	112.86 (15)	N1—C9—H92	108.3
C9—N1—C2	109.26 (16)	C10—C9—H92	108.3
C4—N1—C2	109.93 (16)	H91—C9—H92	107.4
C9—N1—Cr1	112.63 (12)	C11—C10—C9	119.81 (16)
C4—N1—Cr1	108.12 (12)	C11—C10—H10	107.4
C2—N1—Cr1	103.64 (11)	C9—C10—H10	107.4
C8—N2—C6	110.51 (16)	C11—C10—H10B	107.4
C8—N2—C11	108.81 (16)	C9—C10—H10B	107.4
C6—N2—C11	112.46 (16)	H10—C10—H10B	106.9
C8—N2—Cr1	104.29 (12)	N2—C11—C10	115.79 (17)
C6—N2—Cr1	108.67 (12)	N2—C11—H11	108.3
C11—N2—Cr1	111.80 (12)	C10—C11—H11	108.3
O2—C1—O1	123.65 (19)	N2—C11—H11B	108.3
O2—C1—C2	120.98 (19)	C10—C11—H11B	108.3
O1—C1—C2	115.36 (16)	H11—C11—H11B	107.4
N1—C2—C1	109.28 (15)	O10—Ag1—O9	168.15 (6)
N1—C2—H21	109.8	O10—Ag1—O11	97.34 (7)
C1—C2—H21	109.8	O9—Ag1—O11	92.08 (7)
N1—C2—H22	109.8	O10—Ag1—O6	103.11 (7)
C1—C2—H22	109.8	O9—Ag1—O6	86.18 (6)
H21—C2—H22	108.3	O11—Ag1—O6	75.40 (6)
O4—C3—O3	123.7 (2)	Ag1—O9—H1O1	93 (3)
O4—C3—C4	119.96 (19)	Ag1—O9—H2O1	105 (3)
O3—C3—C4	116.29 (16)	H1O1—O9—H2O1	103 (3)
N1—C4—C3	113.23 (15)	Ag1—O10—H1O2	128 (3)
N1—C4—H41	108.9	Ag1—O10—H2O2	111 (3)
C3—C4—H41	108.9	H1O2—O10—H2O2	104 (3)
N1—C4—H42	108.9	Ag1—O11—H1O3	103 (3)
C3—C4—H42	108.9	Ag1—O11—H2O3	113 (3)
H41—C4—H42	107.7	H1O3—O11—H2O3	107 (3)
O6—C5—O5	123.8 (2)		
			
Cr1—O1—C1—O2	−174.74 (17)	C11—N2—C6—C5	129.15 (18)
Cr1—O1—C1—C2	4.1 (2)	Cr1—N2—C6—C5	4.8 (2)
C9—N1—C2—C1	−157.68 (16)	O6—C5—C6—N2	173.04 (19)
C4—N1—C2—C1	77.96 (19)	O5—C5—C6—N2	−7.8 (3)
Cr1—N1—C2—C1	−37.41 (17)	Cr1—O7—C7—O8	179.2 (2)
O2—C1—C2—N1	−156.79 (19)	Cr1—O7—C7—C8	−3.9 (3)
O1—C1—C2—N1	24.3 (2)	C6—N2—C8—C7	84.3 (2)
Cr1—O3—C3—O4	−168.99 (18)	C11—N2—C8—C7	−151.72 (18)
Cr1—O3—C3—C4	11.7 (2)	Cr1—N2—C8—C7	−32.3 (2)
C9—N1—C4—C3	120.00 (18)	O8—C7—C8—N2	−157.0 (2)
C2—N1—C4—C3	−117.75 (18)	O7—C7—C8—N2	25.9 (3)
Cr1—N1—C4—C3	−5.26 (18)	C4—N1—C9—C10	−61.7 (2)
O4—C3—C4—N1	177.0 (2)	C2—N1—C9—C10	175.70 (17)
O3—C3—C4—N1	−3.6 (2)	Cr1—N1—C9—C10	61.1 (2)
Ag1—O6—C5—O5	−62.2 (3)	N1—C9—C10—C11	−39.4 (3)
Ag1—O6—C5—C6	117.0 (2)	C8—N2—C11—C10	177.20 (17)
Cr1—O5—C5—O6	−173.92 (17)	C6—N2—C11—C10	−60.0 (2)
Cr1—O5—C5—C6	6.9 (2)	Cr1—N2—C11—C10	62.6 (2)
C8—N2—C6—C5	−109.04 (19)	C9—C10—C11—N2	−30.0 (3)

### Hydrogen-bond geometry (Å, º)

**Table:**

*D*—H···*A*	*D*—H	H···*A*	*D*···*A*	*D*—H···*A*
O9—H1*O*1···O3^i^	0.84 (1)	1.95 (1)	2.797 (2)	178 (3)
O9—H2*O*1···O8^ii^	0.85 (1)	1.93 (1)	2.767 (3)	172 (4)
O10—H1*O*2···O5^iii^	0.85 (1)	2.02 (1)	2.870 (2)	173 (4)
O10—H2*O*2···O2^iv^	0.85 (1)	1.89 (1)	2.729 (3)	170 (4)
O11—H1*O*3···O7^ii^	0.84 (1)	2.33 (2)	3.142 (3)	163 (4)
O11—H2*O*3···O8^v^	0.83 (1)	1.99 (2)	2.791 (3)	161 (3)

Symmetry codes: (i) −*x*+1/2, −*y*+1, *z*+1/2; (ii) −*x*+1, *y*+1/2, −*z*+1/2; (iii) *x*+1/2, −*y*+1/2, −*z*+1; (iv) *x*+1, *y*, *z*; (v) −*x*+3/2, −*y*+1, *z*+1/2.
